# The association between dietary habits and metabolic syndrome: findings from the Shahedieh-cohort study

**DOI:** 10.1186/s40795-022-00609-5

**Published:** 2022-10-23

**Authors:** Zahra Fallah, Mina Darand, Amin Salehi-Abargouei, Masoud Mirzaei, Gordon A. Ferns, Sayyed Saeid Khayyatzadeh

**Affiliations:** 1 Nutrition and Food Security Research Center, Shahid Sadoughi University of Medical Sciences, Shohadaye gomnam BLD. ALEM square, Yazd, Iran; 2 Department of Nutrition, School of Public Health, Shahid Sadoughi University of Medical Sciences, Yazd, Iran; 3 Department of Clinical Nutrition, School of Nutrition and Food Science, Food Security Research Center, Isfahan University of Medical Sciences, Isfahan, Iran; 4 Yazd Cardiovascular Research Center, Shahid Sadoughi University of Medical Sciences, Yazd, Iran; 5 Brighton & Sussex Medical School, Division of Medical Education, Falmer, Brighton, Sussex, UK

**Keywords:** Metabolic syndrome, Dietary habits, Barbecued-food, Meal, Fried food

## Abstract

**Objective:**

Metabolic syndrome (MetS) is a complex disorder with an increasing prevalence globally. Limited data are available about the association between dietary habits and the prevalence of MetS. The present cross-sectional study aimed to investigate the association between dietary habits and MetS in a large population sample from Iranians.

**Methods:**

The study was conducted on 9261 adults aged 35–70 years who attended the baseline phase of Shahedieh cohort study, Yazd, Iran. Dietary habits including meal frequency, fried food consumption, adding salt to prepared meal, barbecued food consumption, used oil type and reuse oil number were assessed by a standard questionnaire. MetS was defined using the National Cholesterol Education Program Adult Treatment Panel III criteria. Logistic regression was used in different adjusted models to investigate the relationship between dietary habits and MetS: (Model I: adjusted for age, sex and energy. Model II: Model I + adjusted for wealth score index and physical activity. Model III: Model II + adjusted for cardiovascular diseases and liver diseases).

**Results:**

The subjects who ate barbecued-food more than 3 times/ month had 1.18 times greater odds for MetS than individual who ate this less than once/ month (OR: 1.18, 95% CI: 1.01–1.38). After further adjustment for other confounding variables, the association remained significant. No significant association was found between other dietary habits and odds of MetS.

**Conclusion:**

Higher intakes of barbecued-food consumption were related to the prevalence of MetS. Larger longitudinal studies in other population groups are needed to confirm these associations.

## Introduction

Metabolic syndrome (MetS) is a clustering of metabolic and anthropometric abnormalities, including abdominal obesity, hypertension, increased blood glucose, elevated triglyceride (TG) and decreased high-density lipoprotein cholesterol (HDL-c) that is associated with an increased risk of coronary heart disease, stroke, type 2 diabetes and mental disorders [1, 2]. The global prevalence of MetS is increasing dramatically, and its prevalence among adults was approximately 25% in 2017 [3]. The prevalence of MetS among Iranians is high and is estimated to be approximately 30.4% [4]. MetS is a major health care problem and reduces the quality of life, therefore, finding effective strategies to prevent and manage this disease is essential [5, 6].

The pathophysiology of MetS is complex, and genetic as well as environmental factors are involved [7]. Diet is one of the most important modifiable environmental factors [8]. Some studies [9, 10] but not all [11, 12] indicate that poor eating habits can lead to the development and progression of MetS. A recent study indicated that subjects with MetS had a higher consumption of fatty and sweeter food and undesirable eating habits such as faster eating and frequent overeating than healthy people [9]. Adding salt to the food, not regularly eating salads, high meat consumption, a high intake of fried foods and adherence to the western diets are associated with risk of MetS [10, 12]. However, limited data are available about some other dietary habits including meal frequency or barbecued food consumption and MetS. Therefore, the aim of this study was evaluating the relationship between some dietary habits and prevalence of MetS among a large sample of adults who live in central region of Iran.

## Method and materials

### Study population and data collection

The present study is a cross-sectional analysis of subjects in the recruitment phase of the Shahedieh cohort study (this study was begun in 2015 and will be completed in 2025), and is a part of the PERSIAN multi-center cohort study performed on a representative sample of the Iranian adult population of 35–70 years old from 2015 to 2016 [13]. The details of the study protocol of the PERSIAN cohort was published previously [14]. In enrollment of this cohort study, 10,000 people 35–70 years residing in three areas of Yazd, Iran were recruited by multistage cluster random. Inclusion criteria included men and women aged 35–70 years that were active and energetic enough to participate in the study, being of Iranian descent and living in the designated areas for at least 9 months of the year. The individuals with physical or psychological disabilities who were unable to complete the enrollment process were excluded from the cohort study. History of diseases was assessed by a physician. In this cross-sectional study, the individuals with a history of cancer or autoimmune diseases were excluded (*n* = 739), and finally 9261 individuals were included in analyses. The written informed consent was obtained from all the participants and the ethics committee of Shahid Sadoughi University of Medical Sciences approved the study (approval code: IR.SSU.SPH.REC.1397.161). All methods were carried out in accordance with relevant guidelines and regulations.

### Demographic assessments

Data on age and sex were obtained through by face to face interview. The participants reported their physical activity level in the last year, and the data obtained from questionnaire was converted into the metabolic equivalent of task hours per week (MET-h/wk) [15]. Wealth score index (WSI) is estimated by multiple correspondence analysis of variables (own car, car type, the number of books the participant has read in the past year, the total number of international trips the participant has taken during lifetime, the number of international pilgrimage trips the participant has taken, the number of international non-pilgrimage trips the participant has taken and the number of trips the participant has made within Iran in the past 10 years).

### Anthropometric and blood pressure measurements

Weight was measured using a digital scale (SECA, model 755, Germany) with minimum clothing and without shoes with an accuracy of 0.1 kg. Height was measured accurately without shoes by a tape measure attached to the wall without any bumps with a precision of 0.5 cm. Body Mass Index (BMI) (kg/m^2^) was calculated as the weight (kg) divided by height squared (m^2^). Mid-distance between the iliac crest and lowest rib was considered to measure waist circumference (WC). Blood pressure (BP) was measured twice by an experienced nurse using a digital automatic blood pressure monitor (Model M6 Comfort; Omron), with a 5-minute interval between readings.

### Dietary intakes assessments

Dietary intakes were assessed using a 121-item semi-quantitative food frequency questionnaire (FFQ), and the participants were asked about their dietary intakes over the past year. The study participants were interviewed by trained nutritionists. Two types of questions were asked from the participants about each food item: 1) the frequency of food consumption (number of times per month, week, or day the food was consumed) in the previous year, and 2) the amount of the food that was usually consumed every time (portion size based on the standard serving sizes commonly consumed by Iranians). All reported intakes were converted to g/day using household measures of consumed foods. Finally, the Nutritionist IV software was used to calculate nutrients intakes [16].

### Dietary habits evaluation

Information on dietary habits was obtained at the same time as blood samples were collected. Dietary habits including meal frequency (< 3 meals/day, 3 meals/day, 4–6 meals/day and >  6 meals/day), fried food consumption (< 1 time/month, 1–3 times/month, 1–3 times/week and daily), adding salt to prepared food (no, sometimes, yes), barbecued food consumption (< 1 time/month, 1–3 times/month and >  3 time/month), used oil type (solid oil, semi solid oil or margarine, liquid oil, frying oil), (none, once, more than twice) were assessed through a standard questionnaire during the past year.

### Biochemical assessment

After 10–12 hours of fasting, 25 ml of the patient’s blood sample was collected. Blood samples were centrifuged to separate serum. Serum fasting blood glucose (FBG), triglyceride (TG), and high-density lipoprotein cholesterol (HDL-c) were measured using an auto-analyzer (Analyzer BT1500) using Pars Azmun standard kits.

### Metabolic syndrome definition

MetS was defined based on National Cholesterol Education Program Adult Treatment Panel III (NCEP ATP III) criteria. According to the NCEP ATP III definition, metabolic syndrome is present if three or more of the following five criteria are met: waist circumference over 40 in. (men) or 35 in. (women), blood pressure equal to or greater than 130/85 mmHg, TG level equal to or greater than 150 mg/dl, HDL-c level less than 40 mg/dl (men) or 50 mg/dl (women) and FBG equal to or greater than 100 mg/dl [17].

### Statistical analysis

The quantitative (age, physical activity, BMI, WC, FBG, and dietary intakes) and qualitative data (gender, cardiovascular or liver diseases presence) were compared across categories for dietary habits using one-way ANOVA and chi-square tests, respectively. Tukey test was applied as a post hoc analysis. To find the association between dietary habits and metabolic syndrome, we used logistic regression in different models. In model I, we adjusted only age and total energy intake; in model II, BMI, gender, smoking, and physical activity were additionally adjusted. Final adjustments were performed for the history of cardiovascular diseases and liver diseases. Data were analyzed using the Statistical Package for Social Sciences (SPSS, version 15.0). *P* values less than 0.05 were considered statistically significant.

## Results

The general characteristics of study participants across categories of dietary habits are reported in Table 1. The findings of Tukey’s analysis show that consumption of barbecued food and fried foods and also, adding salt to prepared meals was more common among young people. Furthermore, the WSI was significantly different among the categories of fried food consumption, barbecued food consumption, and used oil type. For example, people who used more barbecued foods and also used frying oil in food preparation had a higher WSI.

**Table 1:** General characteristics of study participants according to categories of dietary habits

		Age	Sex (Male)	Physical activity	Body mass index	Wealth score index	Cardiovascular Diseases (%)	Liver diseases (%)
Meal frequency	< 3 meal/day	48.39 ± 9.87^a^	182 (49.1)	41.23 ± 8.04	28.24 ± 5.55	−0.404 ± 1.015	23.9	13.9
	3 meal/day	48.82 ± 1.11	801 (53.3)	41.23 ± 7.78	28.29 ± 5.21	−0.201 ± 1.060	25.2	14.7
	4–6 meal/day	48.16 ± 9.46	3535 (50.7)	41.03 ± 7.10	28.41 ± 4.71	0.072 ± 0.964	24.1	15.5
	> 6 meal/day	48.44	144 (54.8)	41.86 ± 7.62	28.41 ± 4.68	0.150 ± 0.970	26	15.5
*P-value*^*b*^		0.109	0.155	0.239	0.781	< 0.001	0.725	0.752
Fried-food consumption	< 1 time/month	54.41 ± 9.65	247 (43.3)	40.22 ± 7.66	28.86 ± 5.08	−0.533 ± 1.088	43	19.2
	1–3 time/month	49.26 ± 9.65	1322 (49.6)	40.91 ± 7.49	28.47 ± 4.4.80	−0.008 ± 0.983	26.4	17.4
	1–3 time/week	47.17 ± 9.26	2550 (53.8)	41.24 ± 7.22	28.25 ± 4.80	0.094 ± 0.973	21.6	14.2
	Daily	47.56 ± 9.15	543 (48.1)	41.33 ± 6.70	28.50 ± 4.90	−0.026 ± 0.942	21.6	13.3
*P-value*		* < 0.001	< 0.001	0.004	0.014	* < 0.001	< 0.001	< 0.001
Adding salt to prepared meal	No	48.73 ± 9.68	3365 (48.1)	40.95 ± 7.13	28.55 ± 4.82	−0.012 ± 0.998	27.3	15.7
	Sometimes	47.27 ± 9.26	502 (58.6)	41.51 ± 7.17	28.14 ± 4.75	0.036 ± 0.984	16	14
	Yes	46.50 ± 9.06	795 (63.2)	41.58 ± 8.07	27.62 ± 4.89	−0.020 ± 0.959	13.8	13.9
*P-value*		* < 0.001	< 0.001	0.004	< 0.001	0.402	< 0.001	0.133
barbecued -food consumption	< 1 time/month	50.49 ± 10.03	1450 (43.5)	40.82 ± 7.15	28.61 ± 4.99	−0.345 ± 0.984	29.9	14.7
	1–3 time/month	47.03 ± 8.98	2308 (53.6)	41.34 ± 7.35	28.25 ± 4.76	0.180 ± 0.918	20.6	15.8
	> 3 time/month	46.95 ± 9.43	904 (61.4)	40.98 ± 7.30	28.26 ± 4.66	0.317 ± 0.979	22.5	15.4
*P-value*		* < 0.001	< 0.001	0.007	0.003	* < 0.001	< 0.001	0.384
Used oil type	Solid oil	50.35 ± 10.18	442 (48.9)	41.37 ± 7.76	28.21 ± 4.75	−0.271 ± 0.966	25.1	13.7
	Semi solid oil (Margarine)	49.17 ± 10.02	370 (48.5)	41.38 ± 7.03	28.03 ± 5.16	−0.281 ± 0.979	24.4	12.8
	Liquid oil	48.34 ± 9.57	888 (50.2)	41.02 ± 7.37	28.71 ± 5.01	0.001 ± 1.031	26.2	18.2
	Frying oil	47.67 ± 9.33	2792 (52.2)	41.04 ± 7.25	28.33 ± 4.75	0.087 ± 0.957	22.9	14.6
*P-value*		< 0.001	0.071	0.412	0.003	* < 0.001	0.028	< 0.001
Reuse oil number	none	48.31 ± 9.65	3955 (51.6)	41.16 ± 7.29	28.33 ± 4.81	0.025 ± 0.989	24.4	15.6
	once	48.23 ± 9.28	602 (49.3)	40.83 ± 7.10	28.60 ± 4.94	−0.088 ± 0.987	23.6	13.8
	more than twice	47.39 ± 9.15	96 (48.2)	40.29 ± 7.03	29.00 ± 4.93	0.080 ± 1.087	23.8	15.8
*P-value*		0.398	0.225	0.091	0.041	0.001	0.836	0.258

*P-value after Posthoc Tukey analysis remained significant

^a^Mean ± standard deviation (SD)

^b^One-Way Anova for quantitative variables and chi-square test for qualitative variables

The distribution of MetS and its components are presented in Table 2. The results of Tukey’s analysis showed that TG levels were higher in people who consumed barbecue food more than 3 times a month compared to those who consumed it less than 1 a month, while HDL-C levels were significantly lower. In addition, significant differences were seen for SBP and WC among categories of adding salt to a prepared meal. Surprisingly, individuals who added salt to prepared food had significantly lower waist circumference and systolic blood pressure.

**Table 2:** Metabolic syndrome and its components in categories of dietary habits

		Metabolic syndrome (%)	Triglyceride	Fasting blood glucose	Systolic blood pressure	Diastolic blood pressure	High density lipoprotein-cholesterol	Waist circumference
Meal frequency	< 3 meal/day	4	166.10 ± 108.572^a^	105.128 ± 37.39	109.01 ± 18.694	67.20 ± 11.294	52.768 ± 10.371	95.18 ± 13.24
	3 meal/day	16.7	165.18 ± 103.392	107.69 ± 42.96	110.93 ± 17.241	68.55 ± 10.850	52.785 ± 12.463	95.68 ± 12.27
	4–6 meal/day	75.8	166.99 ± 104.035	107.24 ± 40.47	109.96 ± 16.748	67.85 ± 10.807	52.672 ± 12.165	96.12 ± 11.26
	> 6 meal/day	3.4	184.07 ± 118.439	114.197 ± 51.42	110.85 ± 17.397	67.44 ± 10.375	51.351 ± 11.764	96.80 ± 11.40
*P-value*^b^		0.115	0.061	0.039	0.108	0.058	0.252	0.172
Fried-food consumption	< 1 time/month	7.8	163.42 ± 90.185	112.83 ± 42.85	112.82 ± 18.004	68.04 ± 10.224	52.975 ± 12.019	97.66 ± 11.88
	1–3 time/month	29.6	889.41 ± 99.165	107.98 ± 41.15	110.05 ± 17.121	68.02 ± 10.668	52.983 ± 12.195	96.64 ± 11.38
	1–3 time/week	50.6	167.37 ± 103.924	106.02 ± 39.41	109.61 ± 16.509	67.92 ± 10.844	52.196 ± 12.015	95.51 ± 11.50
	Daily	11.9	172.18 ± 123.40	109.30 ± 46.64	110.97 ± 17.544	67.69 ± 11.401	53.405 ± 12.719	95.96 ± 11.63
*P-value*		0.004	0.256	< 0.001	< 0.001	0.846	0.004	< 0.001
Adding salt to prepared meal	No	78.8	165.20 ± 99.092	108.01 ± 41.63	110.74 ± 17.216	68.15 ± 10.865	52.853 ± 12.097	96.87 ± 11.65
	Sometimes	8.8	172.47 ± 113.38	105.60 ± 38.93	109.13 ± 16.431	67.62 ± 10.821	52.260 ± 12.488	95.33 ± 11.53
	Yes	12.4	174.29 ± 125.57	105.44 ± 39.77	107.30 ± 15.315	66.92 ± 10.548	51.616 ± 12.265	94.54 ± 12.09
*P-value*		< 0.001	0.006	0.051	* < 0.001	0.001	0.003	* < 0.001
barbecued-food consumption	< 1 time/month	39.1	165.34 ± 104.688	109.544 ± 43.143	111.27 ± 15.573	67.73 ± 10.946	53.189 ± 12.103	96.405 ± 11.901
	1–3 time/month	44.5	166.27 ± 101.703	105.666 ± 38.802	109.27 ± 16.538	67.87 ± 10.747	52.429 ± 12.088	95.821 ± 11.357
	> 3 time/month	16.5	173.83 ± 112.22	107.809 ± 42.881	109.94 ± 16.459	68.55 ± 10.761	51.920 ± 12.474	95.26 ± 11.156
*P-value*		0.002	*0.027	0.001	* < 0.001	0.047	*0.001	0.067
Used oil type	Solid oil	10.26	169.55 ± 98.594	111.463 ± 45.428	111.49 ± 18.201	68.40 ± 10.539	52.932 ± 12.266	95.969 ± 11.895
	Semi solid oil (Margarine)	8.68	152.02 ± 83.708	110.833 ± 49.418	110.26 ± 16.767	67.60 ± 10.640	52.776 ± 11.875	95.808 ± 12.230
	Liquid oil	20.12	168.37 ± 111.740	107.987 ± 40.382	110.92 ± 17.201	68.53 ± 10.794	53.495 ± 12.295	96.311 ± 11.834
	Frying oil	60.92	167.98 ± 105.442	106.241 ± 39.526	109.54 ± 16.683	67.66 ± 10.948	52.316 ± 12.159	95.938 ± 11.262
*P-value*		0.137	0.001	< 0.001	0.001	0.012	0.005	0.646
Reuse oil number	none	84.36	167.31 ± 101.899	107.387 ± 41.055	110.16 ± 17.038	67.91 ± 10.845	52.537 ± 12.142	95.973 ± 11.440
	once	13.42	165.68 ± 120.829	108.514 ± 43.339	109.95 ± 16.499	67.96 ± 10.807	53.132 ± 12.408	96.379 ± 12.034
	more than twice	2.21	169.93 ± 102.410	103.472 ± 30.464	108.97 ± 15.567	68.29 ± 10.253	52.742 ± 11.480	96.908 ± 11.662
*P-value*		0.560	0.820	0.262	0.572	0.882	0.285	0.295

*P-value after Posthoc Tukey analysis remained significant

^a^Mean ± standard deviation (SD)

^b^One-Way Anova for quantitative variables and chi-square test for qualitative variables

Dietary macronutrient and micronutrient intakes of study participants according to categories of dietary habits are shown in Tables 3 and 4, respectively. After Tukey Analysis, the individual in the last categories of meal frequency, fried food consumption, and adding salt to a prepared meal had significantly higher energy intake. There were significant differences in protein, fiber, vitamin A, folate, potassium, and sodium across categories of meal frequency. The individuals with daily consumption of fried food received lower amounts of protein, carbohydrate, and fiber. The intake of fat, folate, and potassium was higher in people who ate barbecued food more than 3 times/month than in other people, while the intake of carbohydrates and sodium was lower in them.

**Table 3:** Dietary macronutrient intakes of participants according to the categories of dietary habits

		Energy intake (Kcal/d)	Protein		Fat		Carbohydrate		Fiber		Sucrose	
Dietary habits			Crude	Energy-adjusted	Crude	Energy-adjusted	Crude	Energy-adjusted	Crude	Energy-adjusted	Crude	Energy-adjusted
Meal frequency	< 3 meal/day	2497 ± 1224.78	63.03 ± 31.93	25.51 ± 4.82	69.28 ± 39.73	28.09 ± 9.26	412.2 ± 219.09	164.1 ± 21.67	20.25 ± 12.74	8.34 ± 2.84	50.26 ± 64.39	19.39 ± 17.18
	3 meal/day	2690 ± 1172.56	71.69 ± 33.63	26.73 ± 4.19	73.32 ± 36.40	27.82 ± 8.16	442.9 ± 210.04	163.5 ± 17.96	23.13 ± 11.22	8.79 ± 2.58	40.37 ± 32.03	15.29 ± 10.3
	4–6 meal/day	2919 ± 1136.28	78.31 ± 32.06	26.92 ± 3.55	82.24 ± 35.84	28.68 ± 7.51	476.8 ± 204.02	162.2 ± 16.45	27.80 ± 11.85	9.7 ± 2.6	42.66 ± 35.24	14.56 ± 8.48
	> 6 meal/day	3482 ± 1378.54^a^	91.67 ± 36.31	26.67 ± 3.8	96.84 ± 40.97	28.4 ± 7.08	578.2 ± 247.84	164.5 ± 16.06	36.55 ± 15.04	10.75 ± 2.84	57.17 ± 45.89	15.94 ± 9.04
*P*-value		* < 0.001	* < 0.001	* < 0.001	< 0.001	0.001	* < 0.001	0.004	* < 0.001	* < 0.001	< 0.001	* < 0.001
Fried-food consumption	< 1 time/month	2269 ± 1015.45	62.5 ± 29.93	27.62 ± 4.32	58.86 ± 32.7	26.37 ± 8.02	380.2 ± 180.61	166.7 ± 17.74	21.88 ± 10.6	9.89 ± 3.34	31.07 ± 24.84	13.83 ± 8.46
	1–3 time/month	2711 ± 1065.11	71.88 ± 29.06	26.69 ± 3.76	74.12 ± 31.9	27.88 ± 7.41	448.5 ± 194.32	164.1 ± 16.63	25.44 ± 11.2	9.55 ± 2.72	40.38 ± 35.68	14.82 ± 10.02
	> 3 time/month	2992 ± 1184.95	80.22 ± 33.89	26.87 ± 3.64	84.77 ± 37.33	28.88 ± 7.68	487.3 ± 212.56	161.7 ± 16.89	27.95 ± 12.4	9.49 ± 2.53	45.11 ± 39.25	15.07 ± 9.24
	Daily	3120 ± 1209.74	82.74 ± 33.83	26.58 ± 3.72	89.86 ± 38.79	29.5 ± 7.92	505.6 ± 218.61	160.5 ± 16.87	29.10 ± 12.8	9.46 ± 2.59	46.43 ± 32.46	15.07 ± 8.75
*P*-value		* < 0.001	< 0.001	* < 0.001	* < 0.001	< 0.001	* < 0.001	* < 0.001	* < 0.001	*0.007	* < 0.001	0.023
Adding salt to prepared meal	No	2790 ± 1118.14	74.83 ± 31.6	26.93 ± 3.75	78.29 ± 35.32	28.53 ± 7.69	456.3 ± 199.99	162.5 ± 16.98	26.54 ± 11.77	9.68 ± 2.7	40.63 ± 34.94	14.54 ± 9.02
	Sometimes	3027 ± 1202.96	80.91 ± 33.39	26.81 ± 3.45	83.35 ± 36.88	28.16 ± 7.75	497.3 ± 220.55	162.9 ± 16.75	27.31 ± 12.77	9.12 ± 2.36	44.85 ± 32.67	15.03 ± 8.87
	Yes	3285 ± 1282.24	86.27 ± 36.57	26.24 ± 3.79	92.01 ± 40.75	28.62 ± 7.64	538.5 ± 230.57	162.6 ± 16.9	29.2 ± 13.83	8.95 ± 2.47	54.99 ± 46.3	16.91 ± 11.23
*P*-value		* < 0.001	* < 0.001	< 0.001	* < 0.001	0.362	* < 0.001	0.870	* < 0.001	< 0.001	* < 0.001	< 0.001
barbecued-food consumption	< 1 time/month	2606 ± 1109.5	69.72 ± 31.54	26.83 ± 3.73	70.26 ± 33.29	27.61 ± 8.19	431.8 ± 201.99	164.4 ± 17.77	24.31 ± 11.43	9.5 ± 2.74	38.28 ± 32.24	14.78 ± 9.75
	1–3 time/month	2967 ± 1128.6	79.02 ± 31.49	26.75 ± 3.62	83.83 ± 34.87	28.82 ± 7.37	484.3 ± 204.41	162.0 ± 16.35	27.65 ± 11.7	9.49 ± 2.56	44.12 ± 37.47	14.86 ± 9.18
	> 3 time/month	3249 ± 1240.86	87.46 ± 35.32	27.04 ± 4.07	94.93 ± 41.69	29.61 ± 7.21	524.1 ± 218.51	160.4 ± 16.37	31.04 ± 13.83	9.69 ± 2.72	50.48 ± 42.87	15.4 ± 9.08
*P*-value		* < 0.001	* < 0.001	0.035	* < 0.001	* < 0.001	* < 0.001	* < 0.001	* < 0.001	0.030	* < 0.001	0.090
Type of frying vegetables	Little frying, Sauteing	2864 ± 1149.78	76.47 ± 32.7	26.75 ± 3.73	80.61 ± 36.81	28.58 ± 7.57	468.3 ± 203.78	162.6 ± 16.72	27.1 ± 12.27	9.61 ± 2.64	42.84 ± 34.44	15.06 ± 9.31
	Medium amount of frying, until golden yellow	2946 ± 1200.07	78.91 ± 34.05	26.62 ± 3.78	83.3 ± 36.97	28.92 ± 7.75	480.4 ± 216.57	161.6 ± 17.05	26.93 ± 12.24	9.3 ± 2.62	43.9 ± 41.35	14.79 ± 9.5
	A lot of frying, until browned	3049 ± 1204.55	81.9 ± 34.01	26.95 ± 3.83	85.88 ± 38.21	28.69 ± 8.06	497.1 ± 217.58	161.8 ± 17.77	27.91 ± 13.92	9.22 ± 3.7	46.35 ± 31.95	15.48 ± 9.23
	Does not fry foods	2800 ± 1131.61	75.2 ± 31.27	27.02 ± 3.69	77.24 ± 35.18	28.06 ± 7.65	461.0 ± 204.07	163.5 ± 16.81	26.59 ± 11.5	9.7 ± 2.66	41.54 ± 37.23	14.69 ± 9.39
*P*-value		> 0.001	* < 0.001	0.001	> 0.001	0.001	> 0.001	0.001	0.037	* < 0.001	0.004	0.116
Used oil type	Solid oil	2883 ± 1224.45	75.53 ± 35.13	26.22 ± 3.93	80.73 ± 35.72	28.85 ± 8	472.0 ± 224.73	161.9 ± 17.59	25.8 ± 12.29	9.07 ± 2.52	44.51 ± 44.08	15.38 ± 10.76
	Semi solid oil)Margarine)	2907 ± 1162.38	75.88 ± 32.84	26.1 ± 3.72	83.47 ± 38.71	29.3 ± 8.4	471.2 ± 205.11	161.0 ± 18.12	25.54 ± 11.57	8.94 ± 2.44	44.42 ± 33.2	15.41 ± 9.56
	Liquid oil	2786 ± 1122.41	74.65 ± 30.69	27 ± 3.87	78.24 ± 37.08	28.49 ± 7.96	456.2 ± 201	162.7 ± 17.65	26.78 ± 12.44	9.78 ± 2.8	41.75 ± 39.86	14.73 ± 9.35
	Frying oil	2914 ± 1159.92	78.3 ± 32.84	26.96 ± 3.63	81 ± 35.43	28.31 ± 7.43	477.9 ± 208.44	162.9 ± 16.38	27.36 ± 12.1	9.55 ± 2.59	43.18 ± 35.11	14.89 ± 9.18
*P*-value		0.001	> 0.001	> 0.001	0.004	0.004	0.002	0.017	< 0.001	< 0.001	0.196	0.148
Reuse oil number	none	2866 ± 1139.8	76.62 ± 31.93	26.85 ± 3.74	80.48 ± 36.14	28.58 ± 7.71	468.7 ± 204.19	162.4 ± 17	26.94 ± 12.03	9.56 ± 2.67	42.8 ± 36.02	14.9 ± 9.17
	once	2939 ± 1286.25	78.14 ± 36.51	26.66 ± 3.68	81.07 ± 39.06	28.13 ± 7.48	483.6 ± 229.87	163.4 ± 16.4	26.91 ± 12.72	9.35 ± 2.56	44.51 ± 42.34	15.18 ± 10.54
	more than twice	3088 ± 1213.16	83.22 ± 37.14	26.77 ± 3.97	84.45 ± 35.25	28.02 ± 8.16	508.1 ± 226.4	163.2 ± 17.96	28.5 ± 14.33	9.19 ± 2.44	41.76 ± 32.85	13.85 ± 9.36
*P*-value		0.005	0.007	0.233	0.285	0.108	0.003	0.138	0.195	0.007	0.284	0.163

*P-value after Posthoc Tukey analysis remained significant

^a^Obtained from One-Way Anova

**Table 4:** Dietary micronutrient intakes of participants according to categories of dietary habits

Dietary habits		Calcium		Vitamin A		Folate		Potassium		Sodium	
		Crude	Energy-adjusted	Crude	Energy-adjusted	Crude	Energy-adjusted	Crude	Energy-adjusted	Crude	Energy-adjusted
Meal frequency	< 3 meal/day	809.6 ± 40.3.92	308.6 ± 92.42	572.6 ± 453.89	220.4 ± 148.19	334.5 ± 221.21	137.69 ± 57.13	2933 ± 2021.25	1199 ± 406.33	3746 ± 1871.34	1582 ± 572.34
	3 meal/day	897.5 ± 386.23	319.3 ± 79.22	655.1 ± 455.47	235.6 ± 132.12	359.0 ± 186.75	137.61 ± 49.47	3102 ± 1436.51	1198 ± 376.52	4139 ± 2109.67	1584 ± 548.29
	4–6 meal/day	983.2 ± 411.95	335.0 ± 83.53	773.3 ± 525.03	267.9 ± 160.03	406.0 ± 195.57	142.09 ± 46.59	3631 ± 1558.44	1277 ± 357.05	4354 ± 1979.65	1523 ± 457.15
	> 6 meal/day	1171 ± 478.53^a^	345.8 ± 94.45	945.4 ± 757.19	280.3 ± 177.2	528.3 ± 236.01	156.44 ± 52.68	4835 ± 2013.63	1426 ± 371.86	5020 ± 2295.73	1484 ± 497.77
*P*-value		* < 0.001	< 0.001	* < 0.001	* < 0.001	* < 0.001	* < 0.001	* < 0.001	* < 0.001	< 0.001	* < 0.001
Fried-food consumption	< 1 time/month	750.2 ± 391.25	328.8 ± 95.80	485.4 ± 333.65	221.2 ± 153.33	315.1 ± 166.94	142.23 ± 52.54	2890 ± 1486.17	1313 ± 440.33	3568 ± 2023.5	1607 ± 625.54
	1–3 time/month	858.8 ± 377.38	323.6 ± 89.49	636.0 ± 500.19	240.7 ± 153.83	369.7 ± 178.25	139.8 ± 47.96	3347 ± 1473.89	1267.44 ± 366.54	3994 ± 1713.05	1520 ± 461.05
	> 3 time/month	942.3 ± 422.40	320.2 ± 81.70	724.1 ± 507.80	249.4 ± 143.12	416.1 ± 208.47	141.9 ± 47.24	367 ± 1643.19	1260 ± 356.04	4491 ± 2112.52	1534 ± 482.48
	Daily	972.9 ± 415.87	318.0 ± 83.29	694.3 ± 467.82	230.7 ± 138.11	437.6 ± 198.43	143.9 ± 47.34	3825 ± 1672.71	1257 ± 357.55	4689 ± 2058.53	1532 ± 425.29
P-value		< 0.001	0.013	* < 0.001	*0.025	< 0.001	0.074	< 0.001	0.011	* < 0.001	*0.001
Adding salt to prepared meal	No	984.8 ± 450.08	303.6 ± 81.14	794.1 ± 543.99	234.4 ± 141.27	389.9 ± 193.78	142.8 ± 48.51	3486 ± 1561.91	1282 ± 369.75	4090 ± 1890.79	1506 ± 470.15
	Sometimes	942.3 ± 406.05	315.5 ± 76.8	674.0 ± 464.59	228.6 ± 132.84	409.7 ± 185.78	138.5 ± 42.7	3585 ± 1614.84	1213 ± 336.65	4626 ± 2001.94	1563 ± 440.05
	Yes	892.6 ± 402.67	326.3 ± 87.32	669.6 ± 492.90	247.0 ± 150.65	441.1 ± 227.91	136.9 ± 46.89	3888 ± 1797	1207 ± 349.45	5335 ± 2326.11	1674 ± 533.26
*P*-value		0.061	< 0.001	0.026	< 0.001	* < 0.001	< 0.001	* < 0.001	< 0.001	* < 0.001	* < 0.001
barbecued-food consumption	< 1 time/month	830.5 ± 586.31	321.7 ± 87.97	553.4 ± 389.71	219.3 ± 134.49	357.7 ± 179.73	141.0 ± 47.9	3142 ± 1411.59	1248 ± 372.55	4022 ± 1932.73	1585 ± 475.31
	1–3 time/month	933.4 ± 396.79	320.0 ± 81.72	714.2 ± 459.46	249.1 ± 140.93	407.7 ± 189.23	140.5 ± 46.43	3644 ± 1533.56	1260 ± 352.96	4406 ± 1995.73	1514 ± 464.79
	> 3 time/month	1032 ± 450.15	325.0 ± 88.96	886.1 ± 703.43	281.1 ± 181.07	465.9 ± 241.88	145.7 ± 51.33	4200 ± 1942.45	1318 ± 379.06	4696 ± 2160.26	1480 ± 523.54
*P*-value		< 0.001	* < 0.001	< 0.001	< 0.001	< 0.001	*0.001	< 0.001	* < 0.001	* < 0.001	* < 0.001
Type of frying vegetables	Little frying, Sauteing	909.4 ± 411.26	322.0 ± 84.16	693.4 ± 499.53	248.0 ± 149.48	395.9 ± 190.42	141.4 ± 47.02	3571 ± 1578.16	1278 ± 359.46	4288 ± 1942.72	1537 ± 465.26
	Medium amount of frying, until golden yellow	914.2 ± 426.11	317.1 ± 86.48	693.3 ± 489.58	244.1 ± 147.05	407.5 ± 20.73	141.03 ± 51.02	3543 ± 1998.15	1236 ± 364.87	4426 ± 2235.55	1532 ± 515.64
	A lot of frying, until browned	917.7 ± 410.80	306.0 ± 84.58	743.2 ± 552.92	248.3 ± 153.07	412.8 ± 201.06	138.6 ± 47.01	3652 ± 1702.17	1225 ± 359.61	4541 ± 1978.4	1530 ± 448.89
	Does not fry foods	906.0 ± 400.40	330.6 ± 86.85	640.6 ± 484.04	236.8 ± 145.05	391.6 ± 190.1	142.9 ± 46.49	3504 ± 1615.38	1284 ± 371.36	4190 ± 1923.49	1535 ± 478.81
*P*-value		0.835	* < 0.001	> 0.001	0.014	*0.005	0.106	0.088	> 0.001	* < 0.001	0.980
Used oil type	Solid oil	867.6 ± 416.57	307.7 ± 83.88	622.1 ± 444.36	226.3 ± 151.45	390.5 ± 195.63	138.5 ± 44	3392 ± 1620.3	1209 ± 348. 18	4288 ± 1942.72	1530 ± 584.58
	Semi solid oil)Margarine)	871.7 ± 390.60	303.2 ± 78.06	610.1 ± 427.14	213.6 ± 122.09	373.2 ± 173.64	131.7 ± 38.62	3325 ± 1442.44	1180 ± 343.3	4426. ± 2235.55	1557 ± 487.59
	Liquid oil	896.7 ± 408.42	329.1 ± 91.37	699.6 ± 572.96	255.7 ± 165.38	404.5 ± 220.78	147.9 ± 53.86	3553 ± 1688.05	1305 ± 385.66	4541 ± 1978.4	1557 ± 478.72
	Frying oil	924.5 ± 408.58	323.0 ± 83.23	686.36 ± 478.02	242.8 ± 138.70	400.1 ± 191.59	140.3 ± 45.84	3592 ± 1575.24	1264 ± 356.6	4190 ± 1923.49	1530 ± 463.99
*P*-value		< 0.001	< 0.001	< 0.001	< 0.001	0.001	> 0.001	> 0.001	> 0.001	0.044	0.116
Reuse oil number	none	911.5 ± 406.73	324.1 ± 86.39	685.7 ± 505.69	246.1 ± 150.82	398.6 ± 198.91	142.0 ± 48.2	3551 ± 1598.2	1271 ± 367.41	4283 ± 1995.36	1530 ± 481.01
	once	903.3 ± 435.23	311.2 ± 82.50	656.9 ± 457.64	229.6 ± 130.28	394.4 ± 187.94	138.2 ± 43.67	3521 ± 1633.9	1236 ± 343.77	4433 ± 2117.94	1554. ±475.56
	more than twice	908.2 ± 410.96	320.1 ± 87.25	674.9 ± 498.59	243.6 ± 145.2	426.4 ± 242	138.0 ± 50.27	3688 ± 1744.2	1210 ± 378.4	4683 ± 2061.02	1553 ± 483.96
*P*-value		0.473	< 0.001	0.038	< 0.001	0.109	0.018	0.387	0.001	0.002	0.226

*P-value after Posthoc Tukey analysis remained significant

^a^Obtained from One-Way Anova

Multivariate-adjusted odds ratios of the associations between dietary habits and metabolic syndrome are presented in Table 5. Participants who ate barbecued food more than three times a month had a greater odds of MetS than those who ate barbecued food less than once a month (OR: 1.185, 95% CI: 1.015–1.383, *P* = 0.051). These associations remained significant after adjustment for for age, sex, energy, WSI and physical activity (OR: 1.200, 95% CI: 1.025–1.405, *P* = 0.028). Further adjustment for cardiovascular and liver diseases did not change the association. (OR: 1.190, 95% CI: 1.014–1.396, *P* = 0.034). No significant associations were observed between odds of MetS with other dietary habits such as meal frequency (OR: 1.296, 95% CI: 0.886–1.895, *P* = 0.85), fried food consumption (OR: 0.902, 95% CI: 0.708–1.150, *P* = 0.771), adding salt to prepared food (OR: 1.011, 95% CI: 0.865–1.180, *P* = 0.781), used oil type (OR: 1.096, 95% CI: 0.932–1.288, *P* = 0.268), and reuse oil number (OR: 1.051, 95% CI: 0.761–1.451, *P* = 0.764) in model I, II and III.

**Table 5:** Multivariable-adjusted odds ratio of the associations between dietary habits and metabolic syndrome

		Model I	Model II	Model III
Meal frequency	< 3 meal/day	1	1	1
	3 meal/day	1.048(0.791–1.387)	1.047(0.790–1.387)	1.030(0.775–1.370)
	4–6 meal/day	0.995(0.768–1.289)	0.995(0.767–1.293)	0.979(0.752–1.274)
	> 6 meal/day	1.296(0.886–1.895)	1.304(0.888–1.914)	1.267(0.859–1.871)
*P-trend*		0.850	0.883	0.931
Fried-food consumption	< 1 time/month	1	1	1
	1–3 time/month	0.928(0.750–1.147)	0.951(0.768–1.179)	1.008(0.810–1.255)
	1–3 time/week	0.973(0.792–1.196)	0.998(0.810–1.229)	1.071(0.865–1.325)
	Daily	0.902(0.708–1.150)	0.921(0.721–1.176)	0.999(0.779–1.281)
*P-trend*		0.771	0.851	0.683
Adding salt to prepared meal	No	1	1	1
	Sometimes	1.049(0.878–1.254)	1.057(0.884–1.264)	1.137(0.949–1.362)
	Yes	1.011(0.865–1.180)	1.010(0.864–1.181)	1.098(0.937–1.286)
*P-trend*		0.781	0.769	0.143
barbecued -food consumption	< 1 time/month	1	1	1
	1–3 time/month	1.034(0.923–1.158)	1.063(0.946–1.194)	1.073(0.953–1.207)
	> 3 time/month	1.185(1.015–1.383)	1.200(1.025–1.405)	1.190(1.014–1.396)
*P-trend*		0.051	0.028	0.034
Used oil type	Solid oil	1	1	1
	Semi solid oil (Margarine)	0.923(0.739–1.154)	0.932(0.745–1.166)	0.914(0.727–1.149)
	Liquid oil	1.033(0.859–1.241)	1.050(0.872–1.265)	0.981(0.811–1.186)
	Frying oil	1.096(0.932–1.288)	1.111(0.943–1.309)	1.072(0.906–1.267)
*P-trend*		0.268	0.206	0.418
Reuse oil number	none	1	1	1
	once	1.077(0.939–1.235)	1.062(0.925–1.219)	1.083(0.941–1.247)
	more than twice	1.051(0.761–1.451)	1.013(0.732–1.402)	1.007(0.721–1.405)
*P-trend*		0.764	0.939	0.969

Model I: adjusted for age, sex and energy

Model II: Model I + adjusted for WSI and physical activity

Model III: Model II + adjusted for cardiovascular diseases and liver diseases

## Discussion

In the present cross-sectional study we found that the greater consumption of barbecued-food was positively associated with greater odds of MetS among Iranian adults. However, there was no significant association between meal frequency, fried food consumption**,** used oil type**,** reuse of oil number**,** and adding salt to prepared food with MetS.

Our findings showed that the consumption of barbecued-food increased the odds of MetS. The studies investigating the association between barbecued-food consumption with MetS and each of its components are scarce. In a study that conducted by Liu et al. [18], higher consumption of barbecued-red meat increased the risk of type 2 diabetes by 1.23 times. It has been found that chemicals produced by cooking meat at high temperatures (grilling/barbecuing) can induce inflammation, oxidative stress, and insulin resistance, and consequently lead to damage of the inner vascular wall and development of atherosclerosis. All of these processes increase the risk of developing high blood pressure and the likelihood of MetS [19]. In contrast, Heller et al. [20] examined the effect of daily consumption of 8 oz. of barbecued-hamburgers and 6 oz. of barbecued-steak on lipid profile of adults. The results of this study showed that daily consumption of barbecued-meat for 15 days increased HDL-c and decreased total cholesterol and low density lipoprotein-cholesterol (LDL-c). However, the short duration of the study, failure to adjust potential confounders, and the small number of participants were major limitations that can not be relied on the results of this study. Some hazardous chemicals such as heterocyclic aromatic amines (HAAs) and polycyclic aromatic hydrocarbons (PAHs) are produced in foods prepared at high temperatures (barbecuing/grilling) [21–23]. These chemicals can induce pro-inflammatory cytokines, decrease insulin secretion, and consequently increase the risk of diabetes and metabolic syndrome [24, 25]. In addition, cooking meat at high temperatures produces advanced glycation end products (AGEs) that are associated with oxidative stress, inflammation, and insulin resistance in animals and humans, which in turn provide the basis for metabolic disorders [26, 27]. More investigations are needed to clarify the mechanistic pathways linking high-temperature cooking with metabolic syndrome.

We found no association between adding salt to prepared food and MetS. In contrast, a study that conducted by Sarebanhassanabadi et al. [10] showed that eating salt with food increases the risk of MetS. Other studies have also reported an association between sodium intake/excretion with food and MetS and its components [28, 29]. A possible reason for this discrepancy is the differences in methods of evaluating salt intake. Baudrand et al. [28, 29] and Oh et al. [28, 29] used urinary sodium (that has higher accuracy compared to the FFQ) to estimate the sodium intake. In addition, differences in design of the study, sample size and mean age of participants are other possible reasons for this discrepancy. The studies investigating the association between fried food consumption and risk of MetS are scarce. Similar to our findings, Sayon-Orea et al. [30] showed no significant association between fried food consumption with prevalence of MetS. However, some studies have reported that eating fried foods increases the insulin resistance and risk type 2 diabetes [31, 32]. The studies with contradictory results [31, 32] did not investigate the association between fried foods intake with incidence or odds of MetS and had different hypotheses, objectives and study design.

The present cross-sectional study has some strengths and limitations that should be considered. First, the present study comprised a large sample size that reduces the bias in interpreting the results. Second, in data analysis, we used different adjustment models to adjust potential confounding factors such as age and gender. However, in the design of cross-sectional, it is not possible to measure causal relationships. In addition, because FFQ is a memory-based dietary assessment method, it may cause some errors in reporting precise intakes.

In conclusion, the present study demonstrated a direct association between barbecued food consumption with the odds of MetS. On the other hand, we did not find any significant relationship between meal frequency, fried food consumption, adding salt to prepared food, used oil type**,** and reuse oil number with odds of MetS. To confirm these relationships, further investigations especially prospective cohort studies are needed.

## Data Availability

The data and materials of the current study is available from the corresponding author on reasonable request.
